# Ex Vivo Expanded Human Vγ9Vδ2 T-Cells Can Suppress Epithelial Ovarian Cancer Cell Growth

**DOI:** 10.3390/ijms20051139

**Published:** 2019-03-06

**Authors:** Tsui Lien Mao, Carol H. Miao, Yi Jen Liao, Ying Jen Chen, Chia Yu Yeh, Chao Lien Liu

**Affiliations:** 1 Department of Pathology, National Taiwan University Hospital, Taipei 100, Taiwan; tlmao@ntu.edu.tw; 2 Department of Pathology, College of Medicine, National Taiwan University, Taipei 100, Taiwan; 3 Center for Immunity and Immunotherapies, Seattle Children’s Research Institute, Seattle, WA 98101, USA; carol.miao@seattlechildrens.org; 4 School of Medical Laboratory Science and Biotechnology, College of Medical Science and Technology, Taipei Medical University, Taipei 11031, Taiwan; yjliao@tmu.edu.tw (Y.J.L.); B614101078@tmu.edu.tw (C.Y.Y.); 5 Division of General Internal Medicine and Geriatrics, Department of Internal Medicine, Chang Gung Memorial Hospital, Taoyuan 33305, Taiwan; ma2875@cgmh.org.tw; 6 Chang Gung University College of Medicine, Taoyuan 33305, Taiwan

**Keywords:** Vγ9Vδ2-T cells, pamidronate (PAM), ovarian cancer, NSG mice, xenograft

## Abstract

γδ-T-cells have attracted attention because of their potent cytotoxicity towards tumors. Most γδ-T-cells become activated via a major histocompatibility complex (MHC)-independent pathway by the interaction of their receptor, Natural Killer Group 2 Member D (NKG2D) with the tumor-specific NKG2D ligands, including MHC class I-related chain A/B (MICA/B) and UL16-binding proteins (ULBPs), to kill tumor cells. However, despite their potent antitumor effects, the treatment protocols specifically targeting ovarian tumors require further improvements. Ovarian cancer is one of the most lethal and challenging female malignancies worldwide because of delayed diagnoses and resistance to traditional chemotherapy. In this study, we successfully enriched and expanded γδ-T-cells up to ~78% from peripheral blood mononuclear cells (PBMCs) with mostly the Vγ9Vδ2-T-cell subtype in the circulation. We showed that expanded γδ-T-cells alone exerted significant cytotoxic activities towards specific epithelial-type OVCAR3 and HTB75 cells, whereas the combination of γδ-T cells and pamidronate (PAM), a kind of aminobisphosphonates (NBPs), showed significantly enhanced cytotoxic activities towards all types of ovarian cancer cells in vitro. Furthermore, in tumor xenografts of immunodeficient NSG mice, γδ-T-cells not only suppressed tumor growth but also completely eradicated preexisting tumors with an initial size of ~5 mm. Thus, we concluded that γδ-T-cells alone possess dramatic cytotoxic activities towards epithelial ovarian cancers both in vitro and in vivo. These results strongly support the potential of clinical immunotherapeutic application of γδ-T-cells to treat this serious female malignancy.

## 1. Introduction

Epithelial ovarian cancer (EOC), the most common and lethal type of ovarian cancer, causes 140,000 deaths annually worldwide [1]. The most critical challenge for EOC treatment is tumor recurrence in many patients following chemotherapy; even when patients experience an initial response, the cancer often becomes resistant to further chemotherapy. Herein, we investigated an adoptive immunotherapy for EOC using expanded human Vγ9Vδ2 T-cells that possess innate and multifunctional antitumor activities.

In primates, a minor portion of CD3^+^ T-cells expresses the γ/δ T-cell receptor (TCR) which account for <10% of T-cells in the thymus, spleen, lymph nodes, and peripheral blood; but they are predominant T-cell types in certain epithelial tissues [2]. Most circulating γδ-T-cells express the Vγ9Vδ2 TCR that interacts with non-peptide phosphoantigens (PAgs), such as aminobisphosphonates (NBPs) which are potent inhibitors of the isopentenyl pyrophosphate (IPP)-processing enzyme, farnesyle pyrophosphate synthase, in the mevalonate pathway, thereby leading to the intracellular accumulation of IPP and consequent activation of Vγ9Vδ2 T-cells in a major histocompatibility complex (MHC)-independent manner [3,4]. Moreover, activated human γδ-T-cells produce large amounts of Th1 cytokines including, interferon (IFN)-γ, tumor necrosis factor (TNF)-α [5], and interleukin (IL)-17 [6]. However, the role of IL-17 in the tumor microenvironment remains controversial [7].

These γδ-T-cells retain a combination of innate and adaptive immune cell characteristics, rendering them appropriate for immunotherapy [8,9]. Furthermore, γδ-T-cells have been isolated and identified from tumor-infiltrating lymphocytes (TILs) in various types of cancer, including colorectal, breast, and renal cell carcinomas [10,11], suggesting their cytotoxic activities towards a broad range of related tumors due to the recognition of shared ligands [12]; however, studies investigating ovarian cancer are relatively limited. Importantly, γδ-T-cells selectively kill tumor cells but not normal cells, a characteristic that has raised great interest in exploring their therapeutic potential. Non-obese diabetic (NOD)-scid gamma (NSG) mice, an established strain lacking functional B-cells, T-cells, and natural killer (NK) cells [13], are frequently used for the evaluation of therapeutic approaches for human cancer treatments, including cell-based therapies, humanized antibodies, and immunotherapeutic protocols [14].

Some clinical studies using Vγ9Vδ2 T-cells cytotoxicity towards various tumors are currently in progress [15,16,17]; however, further improvement is needed due to limited efficacy. Therefore, we took this opportunity to define which ovarian cancer types might be suitable targets for γδ-T-cell-based immunotherapy. In the present study, we demonstrated the potent susceptibility of specific epithelial ovarian tumor cells, HTB75 and OVCAR3, to Vγ9Vδ2 T-cell treatment alone induced cytotoxicity, whereas treatment of Vγ9Vδ2 T cells combined with pamidronate (PAM; a kind of NBP) showed significantly enhanced cytotoxicity activities towards all types of ovarian cancer cells in vitro due to the induction of NKG2D ligands following treatment with PAM. Moreover, γδ-T-cells exert cell-mediated cytotoxicity via contact with tumor cells by extended pseudopodia, which, in turn, leads to the phagocytosis and destruction of cancer cells. Morphologically, tumor cells attacked by single or multiple γδ-T-cells appeared swollen and disintegrated with bleb formation. We also demonstrated that the adoptive transfer therapy of Vγ9Vδ2 T-cells completely eliminated tumor cells in OVCAR3 xenografts of NSG mice when the tumor size was <5 mm in diameter. Even when the tumor size reached 8 mm, Vγ9Vδ2 T-cells could suppress further growth of the tumor cells.

## 2. Results

### 2.1. Ex vivo Expansion of Human Vγ9Vδ2 T-Cells from Healthy Donor PBMCs Depends on Co-Stimulation

**Figure 1:**
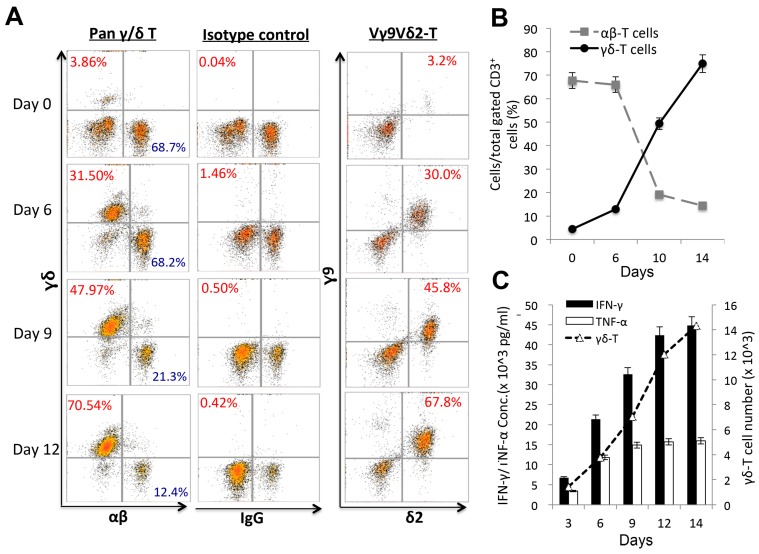
Ex vivo expansion of human peripheral blood mononuclear cell (PBMC)-derived Vγ9Vδ2 T-cells at different time points. Human PBMCs isolated from healthy separate donors were cultured with pamidronate (PAM) and rhIL-2, experiments were performed with many replicates (*n* > 20). (**A**) Expanded percentages (%) of pan-γδ T-cell (**A**, 1st panel), Vγ9Vδ2 T-cell subtypes (**A**, 3rd panel) and the IgG control (**A**, 2nd panel) were evaluated on days 0, 6, 9, and 12 during the culture period (**A**,**B**) using a flow cytometric analysis. (**C**) Cytokine levels (pg/mL) of interferon (IFN)-γ, tumor necrosis factor (TNF)-α, and the γδ-T cell real numbers were detected during the γδ-T-cell expansion period.

PBMCs from healthy donors were isolated and activated with PAM plus IL-2 to enrich γδ-T-cells during the culture period, see Figure 1. Ex vivo expanded γδ-T cells gradually increased and reached a peak level on day 14, which was an approximately 20-fold (78.91%) induction after stimulation compared to normal levels (3.86%) on day 0, see Figure 1A,B. Representative results of expanded γδ-T-cell are shown in Figure 1 with many replicates (*n* > 20). The same expanded cells were then separately stained using Vγ9Vδ2 T-cell markers and showed complete correlation to the percentage increase of expanded pan-γδ-T cells over time, indicating that the expanded pan γδ-T-cells, see Figure 1A, 1st panel, contained the main subtype, Vγ9Vδ2 T-cells, see Figure 1A, 3rd panel, present in human peripheral blood. Moreover, this expansion was accompanied by an induction of the Th1 cytokines, TNF-α and IFN-γ, during the incubation period, see Figure 1C, but not IL-17.

### 2.2. In vitro Cytotoxic Assays to Examine the Destruction of Epithelial Ovarian Tumors by Expanded Vγ9Vδ2 T-Cells Alone

**Figure 2:**
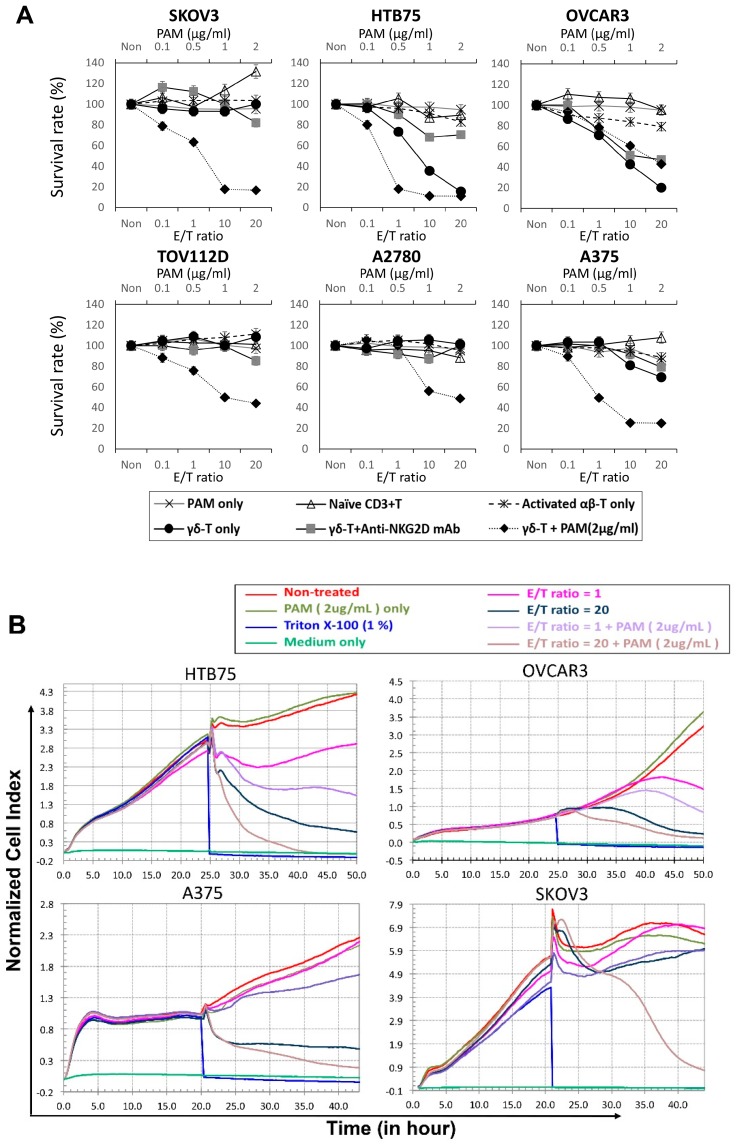
Specific suppression of epithelial ovarian cancer cell lines (HTB75 and OVCAR3), and a melanoma cell line (A375) by expanded Vγ9Vδ2 T-cells alone. (**A**) Standard 24-h cytotoxicity activities were performed with many replicates (*n* > 12) with increasing effector (Vγ9Vδ2 T-cells) concentrations to target E/T ratios of 0, 0.1, 1, 10, and 20 against the cancer cell lines: OVCAR3, HTB75, SKOV3, TOV112D, A2780 and A375. Cytotoxic activities were compared to the Pamidronate (PAM) treated alone, Vγ9Vδ2 T-cells + anti-NKG2D mAb, Vγ9Vδ2 T-cells + PAM (2μg/mL) treated, and activated αβ T-cell- and naïve CD3^+^ T-cell-treated served as the controls of Vγ9Vδ2 T-cells (*n* = 12–20; significance range at * *p* = 0.035 ~ **** *p* < 0.0001). (**B**) Real-time monitoring of cytotoxic activities were compared to the PAM (2μg/mL) treated alone, Vγ9Vδ2 T-cells + PAM (2μg/mL) treated, and Triton X-100 (1%) treated served as the positive control. Real-time monitoring of γδ T- cell-treated alone induced growth inhibition of epithelial-type cells: OVCAR3, HTB75, whereas combination of Vγ9Vδ2-T cells + PAM treatment enhanced growth inhibition of all the cells, including OVCAR3, HTB75, A375 and SKOV3 using the x-CELLigence system. Data are presented as the mean ± SD of three independent experiments.

In order to investigate whether these expanded Vγ9Vδ2 T-cells retained cytotoxic activities towards ovarian cancer cells, cytotoxicity experiments were performed multiple times (*n* = 12–20) using a high-grade serous ovarian carcinoma (HGSC) [18] with epithelial-type tumor cells (HTB75 and OVCAR3), a non-epithelial non-serous ovarian adenocarcinoma (SKOV3) and two endometrioid carcinoma cells (ENOCa; A2780 and TOV112D), whereas a melanoma cell line (A375) was served as the other tumor control. Different titration ratios of healthy donor-derived Vγ9Vδ2 T-cells, Vγ9Vδ2 T-cells treated with NKG2D blocker, activated αβ T-cells and naïve CD3 T-cells (or no T-cells and PAM-treated only as the controls) were added and incubated for 24 h, see Figure 2A, at effector/tumor (E/T) ratios of 0.1, 1, 10, and 20, respectively. HTB75, OVCAR3, and A375 tumor cells were effectively destroyed by Vγ9Vδ2 T-cells at an E/T ratio of 20, as indicated by a reduction in the survival percentage to around 20% (*n* > 12; **** *p* < 0.0001 for HTB75 and OVCAR3) and 60% (*n* > 12; **** *p* < 0.0001 for A375 at 24 h-treated), respectively, compared to the activated αβ T-cell-treated, naive CD3 T-cell-treated and PAM-treated only controls, see Figure 2A, and Table S1. Moreover, the cytotoxic activities of Vγ9Vδ2 T-cells were significantly reduced by an anti-NKG2D mAb blockade, see Figure 2A, and Figure S1B, indicating NKG2D-dependent recognition of tumor cells by γδ T-cells. Interestingly, all six cancers’ cell lines were effectively killed by the combination of Vγ9Vδ2 T-cells and PAM treatment starting from the E/T ratio at 0.1 in vitro compared to the activated αβ T-cell-treated and naïve CD3 T-cell-treated controls (*n* > 12; * *p* = 0.03 ~ **** *p* < 0.0001, Figure 2A and Table S2). Activation of γδ-T-cells was accompanied by the release of IFN-γ and TNF-α into the co-culture medium according to the enzyme-linked immunosorbent assays (ELISAs), see Figure S2. In addition, the ability of γδ T-cells to kill ovarian cancers was also assessed in a co-culture transwell system. Cancer cells and γδ T-cells shared the same medium, but no direct cell–cell interactions were possible due to the physical separation of cells by a polycarbonate membrane. γδ T-cells were unable to exert significant cytotoxicity on cancer cells following incubation. Thus, cancer cells were not susceptible to γδ T-cell non-contact killing.

A real-time cell monitoring system, xCelligence System [19], was used to further investigate the cytotoxic effect of expanded Vγ9Vδ2 T cells towards epithelial ovarian tumor cells. For the first 24 h, tumor cells grew almost directly proportional to the time of culture. The addition of effector cells (γδ-T and naïve CD3^+^T cells) along with the replacement of old medium at 24-h with the E/T ratio of 1, and 20 caused a slight decrease in cell index (CI) of control cells, which was probably related to stress. The addition of E/T ratios at 1 and 20 of γδ-T cells alone resulted in an abrupt decrease in impedance and the CI values were significantly lower in two epithelial-type cells (OVCAR3, and HTB75) compared to each of the control cells, see Figure 2B and Figure S3, and those treated with corresponding E/T ratios of naïve CD3^+^T cells, see Figure S3. Surprisingly, cancer cells treated with γδ-T cells plus PAM showed a significantly enhanced reduction of impedance in both epithelial and non-epithelial-type cells, compared to γδ-T cell treated alone which showed significant cytotoxicity activities not only towards ovarian epithelial-type cells specifically, but also melanoma tumor cells, see Figure 2B. Moreover, PAM-treated alone did not show any significant cell death, whereas Triton-X-100 treated cells all died and served as the positive control, see Figure 2B. These results were consistent with the 3-(4,5-dimethylthiazol-2-yl)-2,5-diphenyltetrazolium bromide (MTT) data, see Figure 2A and Tables S1 and S2, confirming that expanded γδ-T cells treated alone retained significant cytotoxic activities towards epithelial-type ovarian tumors specifically.

### 2.3. Visualization of γδ-T-Cell Contact Cytotoxicity Against Ovarian Cancer OVCAR3 Cells

**Figure 3:**
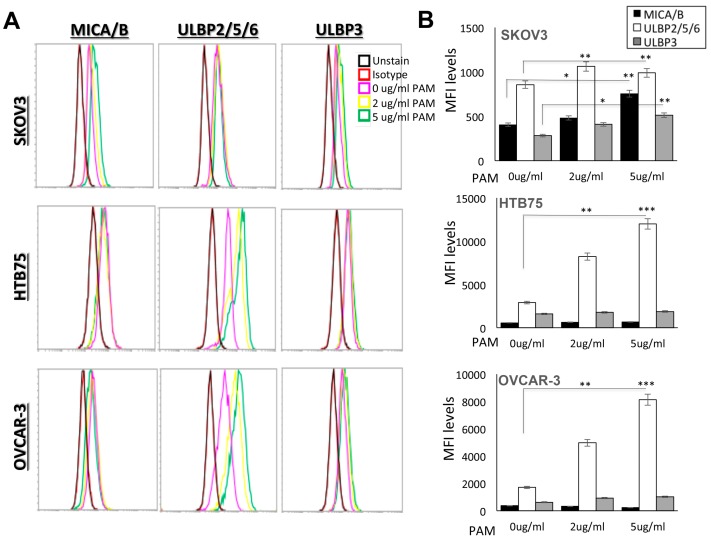
Cell surface expression of NKG2D ligands with and without PAM treatment on tumor cells. (**A**) The indicated tumor cells, SKOV3, HTB75, and OVCAR3, were treated with 2 µg/mL (yellow) and 5 µg/mL (green) PAM for 24 h, and stained with MICA/B, ULBP2/5/6, ULBP3 mAbs or control-Ig (red). Analyses were measured on an Acoustic Focusing flow cytometer using the CellQuest software. (**B**) Fluorescence intensity is shown on a log scale on the X-axis. The numbers indicate median fluorescence intensity (MFI) as an increase in relation to the staining with a control-Ig. Data are presented as the mean ± SD of three independent experiments (* *p* < 0.05; ** *p* < 0.01; *** *p* < 0.001).

Mechanistically, as γδ-T-cells also express the NKG2D receptor, we examined the relative contribution of NKG2D during the γδ-T-cell expansion period on days 0, 6, and 10. Around 90% of expanded γδ-T-cells expressed NKG2D, see Figure S4A, which plays an important role in γδ-T-cells recognition and activation. Moreover, in order to investigate that PAM treatment may enhance γδ-T-cells cytotoxicity, ULBPs and MICA/B, are ligands for human NKG2D; we, therefore, assessed expression levels of MICA/B, ULBP2/5/6, and ULBP3 in the HTB75, OVCAR3, and SKOV3 ovarian cancer cell lines with and without treatment with PAM using a flow cytometric analysis. As shown in Figure S4B, the cell lines without treatment with PAM, OVCAR3 cells showed high levels of MICA/B, ULBP2/5/6, and ULBP3, see Figure S4B, middle panel, whereas HTB75 cells showed high levels of both MICA/B and ULBP2/5/6, see Figure S4B, upper panel; however, SKOV3 cells showed no or minimum expression of all three ligands, see Figure S4B lower panel. For comparison, cell lines were treated with PAM at 2 µg/mL and 5 µg/mL for 24 h; representative results are shown in Figure 3. As can be seen, the expression levels of the two groups of NKG2D ligands varied among the different tumor lines. OVCAR3 and HTB75 cells showed significantly increased ULBPs levels, see Figure 3B; *** *p* < 0.001, whereas SKOV3 showed significantly increased levels of both MICA/B and ULBPs following treatment with PAM compared to the non-treated cells, respectively, see Figure 3B; * *p* < 0.05 and ** *p* < 0.01, respectively.

**Figure 4:**
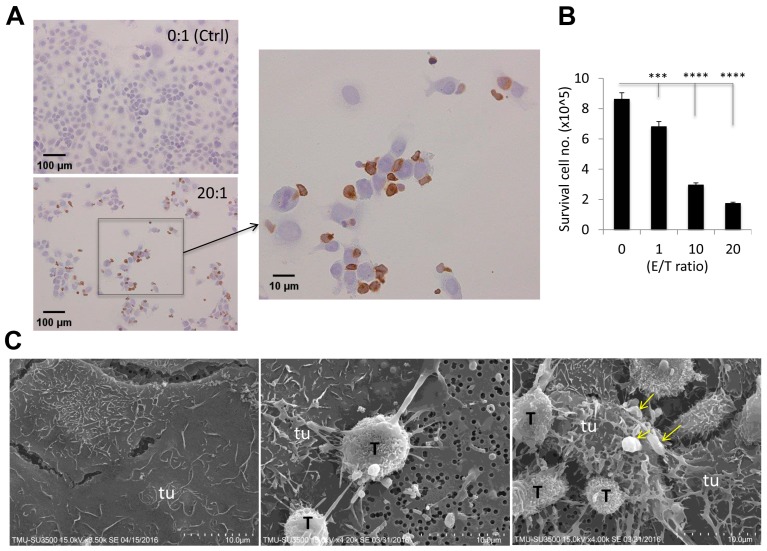
Visualization of γδ-T-cell-attached cytotoxicity towards OVCAR3 cells. γδ-T-cells and OVCAR3 cells were co-cultured at E/T ratios of 0 and 20 on chamber slides. After 24 h, cells were mounted for CD3-IHC staining to illustrate γδ-T-cells (brown color, arrow indicated enlarged area) (**A**), and surviving OVCAR3 cells were counted using light microscopy (**B**). A cytotoxic reaction was carried out with an E/T ratio of 20 in the transwell (**C**), as illustrated by the representative scanning electron microscopic (SEM) images (×1000) of γδ-T-cell (T) cytotoxicity-induced morphologic changes (middle and right) of attacked OVCAR3 cells and the teaming up of γδ-T-cells in the attack as shown in A, using control OVCAR3 (tu) cells only (left) for comparison. Yellow arrows indicated cytoplasmic blebbing. (*** *p* < 0.001; **** *p* < 0.0001).

To further investigate how γδ-T-cells’ contact cytotoxicity acts towards EOC OVCAR3 cells, we set up co-culture cytotoxicity experiments with E/T ratios of 0, 1, 10, and 20 on chamber slides. After 24 h, cells were mounted for CD3 immunohistochemical (IHC) staining, see Figure 4A, and surviving OVCAR3 cell numbers were counted using light microscopy, see Figure 4B. The detailed cytotoxic morphology was examined by light microscopy and further confirmed by scanning electron microscopic (SEM), see Figure 4C. γδ-T-cells and OVCAR3 cells were co-cultured for 24 h in a transwell at an E/T ratio of 20. Specimens were then mounted and subsequently examined using SEM. As shown in Figure 4C, γδ-T-cells (T) were scrambled together all over OVCAR3 (tu) cells by their protruding pseudopodia to form a tight web-like structure, and eventually burrowed into OVCAR3 cells, see Figure 4C, middle and right panels, compared to non-attacked control OVCAR3 cells only, see Figure 4C, left panel. The killed tumor (tu) cells were swollen and disintegrated with cytoplasmic blebbing, see the arrows; Figure 4C, right panel, by a phenomenon known as apoptosis.

### 2.4. Adoptive Transfer of Expanded Vγ9Vδ2 T-Cells Completely Eliminated Smaller-Sized Tumors whereas Modulate the Growth of Large subcutaneous (s.c.) Xenograft Ovarian Tumors in NSG Mice

**Figure 5:**
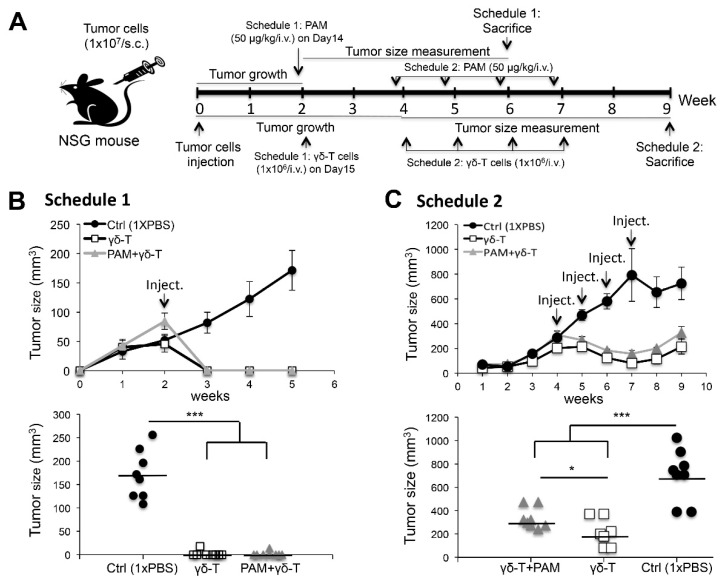
Adoptive transfer of ex vivo activated human Vγ9Vδ2 T-cells together with or without systemic pamidronate (PAM) treatment both showed effective eliminated the growth of subcutaneous (s.c.) human OVCAR3 cell xenografts in NSG mice. (**A**) Two detailed treatment schedules of in vivo study were shown in A. (**B**) In mice treated with schedule 1, the mean tumor size (mm^3^) of eight mice (*n* = 8 per group) during the treatment and follow-up period (upper panel). Tumor size (mm^3^) measured at week six in individual mice in each group (*n* = 8 per group; *** *p* < 0.001; lower panel). (**C**) In mice treated with schedule 2, mean tumor size (mm^3^) of eight mice per group (*n* = 8) were followed during experimental period (upper panel). Tumor size (mm^3^) measured at week 9 in individual mice in each group (*n* = 8 per group; * *p* < 0.05, *** *p* < 0.001; lower panel). Bars represent mean values.

To validate the significantly high cytotoxic activities of Vγ9Vδ2 T-cells toward EOCs in vitro, we needed to overcome the obstacle posed by a lack of natural in vivo counterparts. Hence, we performed human tumor xenografts in NSG [20] mice, which allowed assessment of the antitumor efficacy of adoptively transferred human Vγ9Vδ2 T-cells and support better engraftment compared to any other immunocompromised mouse strain [21]. We selected one of the two EOC cell lines, OVCAR3 cells, which expressed high levels of NKG2D ligands for tumor xenografts and assessed the efficacy of pamidronate (PAM)- and γδ-T-cell-based approaches in NSG mice. As described in Figure 5A, in schedule 1, OVCAR3 cells (10^7^) s.c. implanted into mice grew to form discernible tumor masses (~5 mm in diameter) for two weeks before initiating immunotherapy. To test the antitumor efficacy, we divided the experimental mice into three groups (n = 8/group) which received either a combination of PAM+Vγ9Vδ2 T-cells, Vγ9Vδ2 T-cells alone, or no treatment. The PAM was intravenous (i.v.) injected (50 µg/kg) on day 14 in xenograft NSG mice. After 24 h, expanded human Vγ9Vδ2 T-cells (10^6^) were i.v. injected into mice. In mice treated with schedule 2, OVCAR3 cells (10^7^) were s.c. implanted in mice and allowed to form tumor masses (~8 mm in diameter) for four weeks before initiating immunotherapy. Subsequently, systemic administration of PAM+Vγ9Vδ2 T-cells or Vγ9Vδ2 T-cells alone was repeated weekly for four cycles. Strikingly, mice treated with schedule 1, the growth of OVCAR3 tumors was completely eliminated in mice receiving either systemic PAM+Vγ9Vδ2 T-cells or Vγ9Vδ2 T-cells alone, compared to the control mice group (Ctrl) treated with 1x PBS (*n* = 8/group; Figure 5B upper panel). Individual tumor diameters were measured in each mouse group at week six. In contrast, the tumor size of control mice showed a mean of 8 mm in average diameter (*n* = 8/group; *** *p* < 0.001; Figure 5B lower panel). This result indicated that the Vγ9Vδ2 T-cell infusion yielded an astonishing antitumor response in vivo when the tumor size was relatively small at ~5 mm in diameter. Moreover, in mice treated with schedule 2, a significant and long-term cessation of OVCAR3 tumor growth was achieved compared to control mice in which the tumor size increased over time (*n* = 8/group; Figure 5C upper panel). At week nine, tumor diameters had significantly decreased in both groups of treated mice compared to control mice (*n* = 8/group; * *p* < 0.05, *** *p* < 0.001; Figure 5C lower panel).

**Figure 6:**
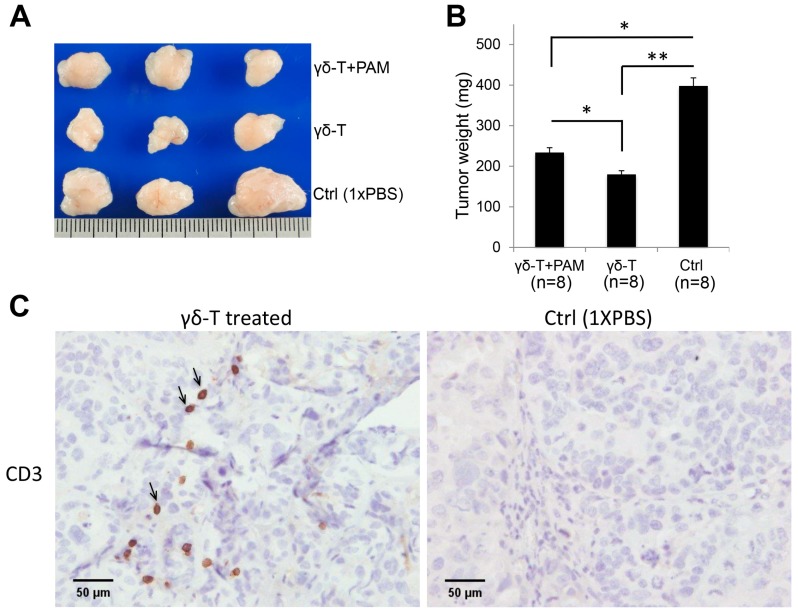
Repeated systemic administration of expanded γδ-T-cell infusions showed significantly enhanced antitumor efficacy of γδ-T-cell therapy in vivo. The endpoint dissection of schedule 2 treated-mice. (**A**) Tumor masses from different mice groups. (**B**) The mean tumor weight (mg) from each treated mouse group (*n* = 8 per group; * *p* < 0.05, ** *p* < 0.01). (**C**) γδ-T-cells confirmed by CD3-IHC staining in tumor sections from different mice groups. CD3 positivity (black arrow) indicated γδ-T-cell infiltration (**C**, left).

Accordingly, the average tumor size, see Figure 6A, and tumor weight, see Figure 6B, showed significant reductions in both the PAM+Vγ9Vδ2 T-cell- and only Vγ9Vδ2 T-cell-treated mouse groups (*n* = 8/group; * *p* < 0.05, ** *p* < 0.01). Apart from this, the experimental mice survived without significant body weight loss or physical disability before the experimental endpoint (tumor mass > 800 mm^3^ in control group). Furthermore, the anti-CD3 IHC analysis revealed the presence of infiltrating human Vγ9Vδ2 T-cells in tumor sections of NSG mice that had received Vγ9Vδ2 T-cells, see Figure 6C.

## 3. Discussion

Tumor-infiltrating γδ-T-cells are composed of Vδ1 and Vδ2 subtypes, which were reported to react to tumors but not to healthy cells. Among these, Vγ9Vδ2 T-cells, a unique subtype in human peripheral blood that showed strong cytotoxicity against cancer cells, including ovarian cancer, may serve as a potent candidate for cancer immunotherapy [22,23]. Here, we showed that activated γδ-T-cells exhibited much higher cytotoxic activities against the EOC cell lines, OVCAR3 and HTB75. This may have partly been due to interactions of the NKG2D receptor on γδ-T-cells with its tumor-expressed ligands, thereby overcoming inhibitory signals by MHC class I molecules [24]. MICA/B [25] and ULBPs [26] are different kinds of NKG2D ligands (NKG2DLs) responsible for Vγ9Vδ2-T cells activation. These ligands are broadly expressed on the surface of various epithelial and hematopoietic tumors [27], and thereby can reinforce antitumor immune responses of Vγ9Vδ2-T cells. Therefore, we also found that 90% of the expanded γδ-T-cells expressed NKG2D, whereas MICA/B, ULBP2/5/6, and ULBP3 were over-expressed by the targeted EOC lines, OVCAR3 and HTB75, to enact the efficient cytotoxic responses by Vγ9Vδ2 T-cells. Interestingly, Vγ9Vδ2 T-cells treated alone specifically suppressed EOCs growth but not in non-epithelial-type SKOV3, TOV112D, and A2780, whereas the combination of Vγ9Vδ2 T-cells and PAM treatment showed significantly enhanced cytotoxic activities towards both epithelial- and non-epithelial-type ovarian cancers starting from the E/T ratio at 0.1 (significance range at * *p* = 0.035 ~ **** *p* < 0.0001; Tables S1 and S2). Different levels of NKG2D ligands expression may explain the differential cytotoxicity of γδ-T cells towards different cancer types. Nevertheless, the effects of combination therapy using Vγ9Vδ2 T-cells plus PAM are uniquely effective towards non-epithelial-type ovarian cancers, as well as enhanced cytotoxicity activities towards other types of cancers, see Figure 2. The addition of PAM may trigger the up-regulation of the expression of tumor-associated antigens MICA/B or ULBPs recognized by γδ-T cells [28]. Indeed, as shown in Figure 3, the expression levels of NKG2D ligands were increased to different levels among the different tumor lines following PAM treatment, which is associated with variable susceptibility of tumor cells to γδ T-cell-mediated lysis. In addition, it was demonstrated that tumor cells released the NKG2D ligands, MICA and MICB, in a soluble form by proteolytic shedding from the tumor cell surface by metalloproteases [29] and protected tumor cells from cytolysis if they were NKG2DL-positive.

Upon contacting with tumor cells, γδ-T-cells extended their pseudopodia and burrowed into target cells as demonstrated by SEM. Also, reactive tumor cells formed spherical protrusions on tumor cell surfaces (so-called “membrane blebbing”). Blebbing, a display of plasmalemmal protrusions, is most commonly associated with cell injury and apoptosis [30,31]. The mechanism of γδ-T-cell-mediated cytotoxicity is likely correlated with cytotoxic T-cells (CTLs) and NK cells that utilize the pore-forming protein, perforin (PFN), to eliminate virus-infected cells and tumor cells [32]. CTLs produce cytotoxic granules in which PFN delivers a group of serine proteases called granzymes to induce target cell apoptosis [33]. However, a detailed understanding of the molecular mechanism of γδ-T-cell-mediated cytotoxicity towards tumors is still needed. In addition, the clinical trials completed to date, particularly those with in vivo γδ-T cells stimulation, have shown only around 10–30% efficacy due to inefficient γδ-T expansion in vivo, as well as the lack of “susceptible” tumor profiles. Such limitations can be circumvented by adoptive T-cell transfer approaches because large amounts of Vγ9Vδ2 T-cells can be expanded and activated ex vivo using clinically feasible protocols [34]. Advantages of the Vγ9Vδ2 T-cell subset include the high efficiency of ex vivo expansion upon short-term culture of PBMCs from healthy donors using Vγ9Vδ2-agonist molecules and rhIL-2, and the lack of alloreactivity of γδ-T-cells [35,36], which can impair autologous Vγ9Vδ2 T-cell expansion. Moreover, our tumor xenografts mouse model showed almost 100% response to the Vγ9Vδ2 T-cells treatment, confirming this approach will be valuable for clinical applications targeting ovarian cancer. In this study, we showed the strong in vitro and in vivo lytic activities of Vγ9Vδ2 T-cells against human EOCs.

An improvement of adoptive γδ-T-cell transfer approaches might be to combine them with NBP treatments, which can sensitize tumor cells to γδ-T-cell recognition. This combined approach was supported by a recent phase I trial in which renal carcinoma patients showed a complete response and their disease pathology was rapidly stabilized [37]. Although we demonstrated that the γδ T-cells + PAM combination treatment caused the susceptibility of tumor cells to γδ T-cell-mediated cytotoxicity via the induction of NKG2D ligands in vitro, our results showed that a systemic injection of PAM 24 h before the adoptive transfer of Vγ9Vδ2 T-cells did not improve therapeutic outcomes compared to Vγ9Vδ2 T-cells alone in vivo. However, PAM addition in the combination therapy showed significantly enhanced cytotoxicity activities in vitro. We, therefore, concluded that the antitumor effect of the regimens of PAM+Vγ9Vδ2 T-cells and Vγ9Vδ2 T-cells alone resides on the effectiveness of Vγ9Vδ2 T-cells due to their effector function. This is also confirmed by the cytotoxic activity of γδ-T cells towards other cancers, such as the melanoma cells in our study. Reports also showed that NBP has the ability to enhance the antitumor efficacy of adoptively transferred Vγ9Vδ2 T-cells [38]. This discrepancy can be explained by three possibilities. First, the antitumor efficacy of adoptively transferred Vγ9Vδ2 T-cells in our study was sufficiently high to overshadow the antitumor effect of NBP, in which the antitumor effect greatly varies from one tumor cell line to another. In tumor cell lines, PAM treatment is associated with an increase in the expression of NKG2D ligands. However, the OVCAR3 cells that we used in the in vivo model already have high levels of NKG2D ligands and, therefore, may be less affected by the PAM treatment. Second, γδ-T-cell responses induced by PAM-treated tumor cells were more sensitive to treatment than those induced by zoledronate-treated cells [39]. Third, the effects and/or conditions of cytotoxicity activities enhancement of γδ-T cells in vitro may not be exactly the same as applications in vivo.

In summary, our study provides strong preclinical evidence for a powerful immunotherapy against EOCs using adoptive Vγ9Vδ2 T-cell transfer. We showed that the EOC, a susceptible tumor type in ovarian cancer, can be effectively treated by γδ-T cells. Importantly, our results indicated that such an approach not only prevented tumor expansion but also completely eradicated preexisting tumors when the tumor size was relatively small in early tumor growth. Furthermore, bisphosphates such as PAM treatment can potentially enhance the therapeutic efficacy by γδ-T-cells transfusion. However, it should be noted that its impact varies with individual tumors, which is tightly associated with the induction of NKG2D ligands. Therefore, for the γδ-T-cells therapeutic applications, it might be beneficial to examine the expression levels of NKG2D ligands in patients’ tumors prior to the choice of whether to combine with PAM treatment or not. In addition, we also evaluated, in detail, the efficacy of targeted cytotoxicity and morphology changes of the tumors and elucidated the mechanisms of tumor eradication. However, although both the size and weight of larger size tumors were significantly reduced by the administration of γδ-T-cells, the complete elimination of large tumors was not achieved. This was probably due to the hypoxic environment within large tumors that might enhance resistance to the infiltrating γδ-T-cell-mediated immune attack. Although the OVCAR3 xenograft model lacked activated αβ T-cells infusion control, which then requires further experimental confirmation, our in vitro cytotoxicity data were precisely indicative of the in vivo results and thereby supported the unique function of γδ T-cells mediated cytotoxicity. A further detailed mechanistic study is required to improve this therapeutic approach.

## 4. Materials and Methods

### 4.1. Ethics, Consent, and Permission

The Taipei Medical University-Joint Institutional Review Board (TMU-JIRB) approved the study (protocol no.: N201605059, 10 June 2016). Informed consent was obtained from each individual healthy volunteer. The research including biohazards, biological agents, toxins, materials or reagents follows the standard biosafety regulations and is reviewed and approved by the Institute’s “Environmental Protection and Biological Safety Committee” (G-104-078, 4 January 2016) before the project started.

### 4.2. Expansion of Human Vγ9Vδ2 T-Cells

Human PBMCs obtained from different healthy individual volunteers were isolated by gradient separation with Ficoll-Paque Plus (GE Healthcare, Chicago, IL, USA), and were cultured in 37 °C with 5% CO_2_ at a density of 1.5 × 10^6^ cells/mL in RPMI 1640 (Invitrogen^TM^, ThermoFisher, Waltham, MA, USA). On the day of isolation, IL-2 (100 U/mL; Peprotech, Rocky Hill, USA), and pamidronate (PAM, 2.0 µg/mL; Peprotech) were added. Additional medium and cytokines were added every 2–3 days over a total culture period of 14 days.

### 4.3. Flow Cytometry

Expanded Vγ9Vδ2 T-cells were purified by positive selection using a human anti-TCR γ/δ T-cell MicroBeads kit (MiltenyiBiotec, Auburn, CA, USA), according to the manufacturer’s instructions. The following monoclonal antibodies (mAbs) were obtained from BD Bioscience (San Jose, CA, USA): FITC-mouse anti-human CD3, PE-mouse anti-human TCRγδ, FITC-mouse anti-human TCRαβ, PE-mouse anti-human Vγ9-TCR, FITC-mouse anti-human Vδ2-TCR, PE-Cy7-mouse anti-human CD314 (NKG2D), PE-mouse anti-human ULBP3, PE-mouse anti-human ULBP2/5/6, APC-mouse anti-human MICA/B, and their IgG controls. After expansion, Vγ9Vδ2 T-cells and tumor cell lines were confirmed by flow cytometry using a FACSCalibur and/or an Attune^®^ NxT Acoustic Focusing cytometer and analyzed using CellQuest Pro (BD Biosciences, San Jose, CA, USA) software version 2.7.873.0.

### 4.4. Tumor Cell Culture

The ovarian cancer cell lines, HTB75, OVCAR3, SKOV3, TOV112D, A2780 and a melanoma cell line A375 were purchased from American Type Culture Collection (ATCC, Manassas, VA, USA) and grown in Dulbecco’s modified Eagle medium (DMEM; Life Technologies, Carlsbad, CA, USA) supplemented with 10% FBS at 37 °C with 5% CO_2_ incubation. All cell lines were tested for species identification, mycoplasma detection and authentication confirmed via short tandem repeat (STR) profile analysis at ATCC.

### 4.5. Cytotoxicity Assays

MTT (Sigma-Aldrich, Saint Louis, MO, USA) cytotoxicity assays were performed in 96-well plates. Tumor cells (10^4^ cells/well) were plated in triplicate wells. After 24 h, one of the NBPs, pamidronate (PAM), at the indicated titration ratios of expanded Vγ9Vδ2 T-cells, activated αβ T-cells and naïve CD3 T-cells at approximate effector/tumor (E/T) ratios were added at different treatment time slots. Where indicated, γδ effector cells were pre-incubated for 30 min with 10μg/mL anti-NKG2D mAb (R/D system, Inc. Minneapolis, MN, USA) before the addition of tumor target cells. MTT values were evaluated using absorbance at 570 nm on an ELISA reader (BioTek, Winooski, VT, USA). The viability of the cells was calculated as the percentage of MTT reduction.

### 4.6. Real-Time Cell Monitoring Using the xCELLigence System

The xCELLigence system was used to monitor cell survival according to the instructions of the supplier (Roche Diagnostics, Mannheim, Germany and ACEA Bioscience, San Diego, CA, USA). Cells were grown overnight and impedance was measured every hour, prior to treatments, as described [40]. Cell impedance is represented by the cell index [CI = (Zi−Z0)/15Ω; where Zi is the impedance at an individual time point and Z0 is background resistance]. A normalized cell index was determined as the cell index at an individual point of time divided by the cell index at the normalization time point.

### 4.7. In Vivo Studies

All mice were kept according to National Institutes of Health guidelines for animal care and the guidelines of the Genomics Research Center, Academia Sinica (Taipei, Taiwan) and maintained in a specific pathogen-free (SPF) facility. The animal use protocol has been reviewed and approved by the Institutional Animal Care and Use Committee (IACUC/IACUP; protocol No: LAC-2015-0251, 22 12 2015). NOD.Cg-PrkdcscidIl2rgtmlWjl/SzJ (NSG) mice were used at 6–10 weeks of age. Mice were randomly assigned to three treatment groups (*n* = 4 mice/group): Ctrl (1x PBS), Vγ9Vδ2 T-cells (10^6^ cells/mouse, by i.v. injection), and PAM plus Vγ9Vδ2 T-cells (50 μg/kg PAM i.v. 1 day before administering Vγ9Vδ2 T-cells (10^6^ cells/mouse) via an i.v. injection).

### 4.8. Visualization of Cell Attachment

After co-culture for 24 h at an E/T ratio of 20, attached cells were imaged using scanning electron microscopy (SEM, Nova NanoSEM 450, FEI, USA) to compare the morphology of OVCAR3 cells on culture transwells (control group). SEM imaging was performed after fixing cells with 2% paraformaldehyde + 2.5% glutaraldehyde (Sigma-Aldrich, USA), dehydrating samples with graded ethanol (EMSURE^®^ Ph Ethanol absolute for analysis), and further drying samples in an oven for 24 h. Fixed samples were sputter-coated with gold and imaged using SEM.

### 4.9. Statistical Analysis

Data were analyzed with SigmaPlot 13.0 software and GraphPad Prism 6. Results are presented as the mean ± standard deviation (SD). The statistical significance of the difference between means was determined using a two-tailed Student’s *t*-test for the intergroup comparisons. Comparisons between groups were determined with a one-way ANOVA analysis. Differences were considered significant at * *p* < 0.05, ** *p* < 0.01, *** *p* < 0.001, **** *p* < 0.0001.

## Supplementary Materials

Supplementary materials can be found at https://www.mdpi.com/1422-0067/20/5/1139/s1.

## References

[1] Kotsopoulos I.C., Papanikolaou A., Lambropoulos A.F., Papazisis K.T., Tsolakidis D., Touplikioti P., Tarlatzis B.C. (2014). Serous ovarian cancer signaling pathways. Int. J. Gynecol. Cancer.

[2] Girardi M. (2006). Immunosurveillance and immunoregulation by gammadelta t cells. J. Investig. Dermatol..

[3] Gober H.J., Kistowska M., Angman L., Jeno P., Mori L., De Libero G. (2003). Human t cell receptor gammadelta cells recognize endogenous mevalonate metabolites in tumor cells. J. Exp. Med..

[4] Kunzmann V., Bauer E., Wilhelm M. (1999). Gamma/delta t-cell stimulation by pamidronate. N. Engl. J. Med..

[5] Correia D.V., d’Orey F., Cardoso B.A., Lanca T., Grosso A.R., deBarros A., Martins L.R., Barata J.T., Silva-Santos B. (2009). Highly active microbial phosphoantigen induces rapid yet sustained mek/erk- and pi-3k/akt-mediated signal transduction in anti-tumor human gammadelta t-cells. PLoS ONE.

[6] Wakita D., Sumida K., Iwakura Y., Nishikawa H., Ohkuri T., Chamoto K., Kitamura H., Nishimura T. (2010). Tumor-infiltrating il-17-producing gammadelta t cells support the progression of tumor by promoting angiogenesis. Eur. J. Immunol..

[7] Van Acker H.H., Anguille S., Van Tendeloo V.F., Lion E. (2015). Empowering gamma delta t cells with antitumor immunity by dendritic cell-based immunotherapy. Oncoimmunology.

[8] Vantourout P., Hayday A. (2013). Six-of-the-best: Unique contributions of gammadelta t cells to immunology. Nat. Rev. Immunol..

[9] Hannani D., Ma Y., Yamazaki T., Dechanet-Merville J., Kroemer G., Zitvogel L. (2012). Harnessing gammadelta t cells in anticancer immunotherapy. Trends Immunol..

[10] Xu C., Zhang H., Hu H., He H., Wang Z., Xu Y., Chen H., Cao W., Zhang S., Cui L. (2007). Gammadelta t cells recognize tumor cells via cdr3delta region. Mol. Immunol..

[11] Lang J.M., Kaikobad M.R., Wallace M., Staab M.J., Horvath D.L., Wilding G., Liu G., Eickhoff J.C., McNeel D.G., Malkovsky M. (2011). Pilot trial of interleukin-2 and zoledronic acid to augment gammadelta t cells as treatment for patients with refractory renal cell carcinoma. Cancer Immunol. Immunother..

[12] Deniger D.C., Maiti S.N., Mi T., Switzer K.C., Ramachandran V., Hurton L.V., Ang S., Olivares S., Rabinovich B.A., Huls M.H. (2014). Activating and propagating polyclonal gamma delta t cells with broad specificity for malignancies. Clin. Cancer Res..

[13] Shultz L.D., Goodwin N., Ishikawa F., Hosur V., Lyons B.L., Greiner D.L. (2014). Human cancer growth and therapy in immunodeficient mouse models. Cold Spring Harb. Protocols.

[14] Shultz L.D., Brehm M.A., Garcia-Martinez J.V., Greiner D.L. (2012). Humanized mice for immune system investigation: Progress, promise and challenges. Nat. Rev. Immunol..

[15] Kobayashi H., Tanaka Y., Yagi J., Osaka Y., Nakazawa H., Uchiyama T., Minato N., Toma H. (2007). Safety profile and anti-tumor effects of adoptive immunotherapy using gamma-delta t cells against advanced renal cell carcinoma: A pilot study. Cancer Immunol. Immunother..

[16] Bonneville M., Scotet E. (2006). Human vgamma9vdelta2 t cells: Promising new leads for immunotherapy of infections and tumors. Curr. Opin. Immunol..

[17] Kunzmann V., Smetak M., Kimmel B., Weigang-Koehler K., Goebeler M., Birkmann J., Becker J., Schmidt-Wolf I.G., Einsele H., Wilhelm M. (2012). Tumor-promoting versus tumor-antagonizing roles of gammadelta t cells in cancer immunotherapy: Results from a prospective phase i/ii trial. J. Immunother..

[18] Anglesio M.S., Wiegand K.C., Melnyk N., Chow C., Salamanca C., Prentice L.M., Senz J., Yang W., Spillman M.A., Cochrane D.R. (2013). Type-specific cell line models for type-specific ovarian cancer research. PLoS ONE.

[19] Ke N., Wang X., Xu X., Abassi Y.A. (2011). The xcelligence system for real-time and label-free monitoring of cell viability. Methods Mol. Biol..

[20] Cao X., Shores E.W., Hu-Li J., Anver M.R., Kelsall B.L., Russell S.M., Drago J., Noguchi M., Grinberg A., Bloom E.T. (1995). Defective lymphoid development in mice lacking expression of the common cytokine receptor gamma chain. Immunity.

[21] Ito M., Hiramatsu H., Kobayashi K., Suzue K., Kawahata M., Hioki K., Ueyama Y., Koyanagi Y., Sugamura K., Tsuji K. (2002). Nod/scid/gamma(c)(null) mouse: An excellent recipient mouse model for engraftment of human cells. Blood.

[22] Kekre N., Antin J.H. (2014). Hematopoietic stem cell transplantation donor sources in the 21st century: Choosing the ideal donor when a perfect match does not exist. Blood.

[23] Davey M.S., Willcox C.R., Hunter S., Kasatskaya S.A., Remmerswaal E.B.M., Salim M., Mohammed F., Bemelman F.J., Chudakov D.M., Oo Y.H. (2018). The human vdelta2(+) t-cell compartment comprises distinct innate-like vgamma9(+) and adaptive vgamma9(-) subsets. Nat. Commun..

[24] Cosman D., Mullberg J., Sutherland C.L., Chin W., Armitage R., Fanslow W., Kubin M., Chalupny N.J. (2001). Ulbps, novel mhc class i-related molecules, bind to cmv glycoprotein ul16 and stimulate nk cytotoxicity through the nkg2d receptor. Immunity.

[25] Nakajima N.I., Niimi A., Isono M., Oike T., Sato H., Nakano T., Shibata A. (2017). Inhibition of the hdac/suv39/g9a pathway restores the expression of DNA damage-dependent major histocompatibility complex class i-related chain a and b in cancer cells. Oncol. Rep..

[26] Dansako H., Imai H., Ueda Y., Satoh S., Wakita T., Kato N. (2018). Ulbp1 is induced by hepatitis c virus infection and is the target of the nk cell-mediated innate immune response in human hepatocytes. FEBS Open Bio..

[27] Carbone E., Neri P., Mesuraca M., Fulciniti M.T., Otsuki T., Pende D., Groh V., Spies T., Pollio G., Cosman D. (2005). Hla class i, nkg2d, and natural cytotoxicity receptors regulate multiple myeloma cell recognition by natural killer cells. Blood.

[28] Shimizu T., Tomogane M., Miyashita M., Ukimura O., Ashihara E. (2018). Low dose gemcitabine increases the cytotoxicity of human vgamma9vdelta2 t cells in bladder cancer cells in vitro and in an orthotopic xenograft model. Oncoimmunology.

[29] Salih H.R., Rammensee H.G., Steinle A. (2002). Cutting edge: Down-regulation of mica on human tumors by proteolytic shedding. J. Immunol..

[30] Babiychuk E.B., Monastyrskaya K., Potez S., Draeger A. (2011). Blebbing confers resistance against cell lysis. Cell Death Differ..

[31] Maeda T., Toyoda F., Imai S., Tanigawa H., Kumagai K., Matsuura H., Matsusue Y. (2016). Lidocaine induces rock-dependent membrane blebbing and subsequent cell death in rabbit articular chondrocytes. J. Orthop. Res..

[32] Froelich C.J., Orth K., Turbov J., Seth P., Gottlieb R., Babior B., Shah G.M., Bleackley R.C., Dixit V.M., Hanna W. (1996). New paradigm for lymphocyte granule-mediated cytotoxicity. Target cells bind and internalize granzyme b, but an endosomolytic agent is necessary for cytosolic delivery and subsequent apoptosis. J. Biol. Chem..

[33] Keefe D., Shi L., Feske S., Massol R., Navarro F., Kirchhausen T., Lieberman J. (2005). Perforin triggers a plasma membrane-repair response that facilitates ctl induction of apoptosis. Immunity.

[34] Kondo M., Sakuta K., Noguchi A., Ariyoshi N., Sato K., Sato S., Sato K., Hosoi A., Nakajima J., Yoshida Y. (2008). Zoledronate facilitates large-scale ex vivo expansion of functional gammadelta t cells from cancer patients for use in adoptive immunotherapy. Cytotherapy.

[35] Schilbach K.E., Geiselhart A., Wessels J.T., Niethammer D., Handgretinger R. (2000). Human gammadelta t lymphocytes exert natural and il-2-induced cytotoxicity to neuroblastoma cells. J. Immunother..

[36] Lamb L.S., Musk P., Ye Z., van Rhee F., Geier S.S., Tong J.J., King K.M., Henslee-Downey P.J. (2001). Human gammadelta(+) t lymphocytes have in vitro graft vs leukemia activity in the absence of an allogeneic response. Bone Marrow Transplant..

[37] Kobayashi H., Tanaka Y., Yagi J., Minato N., Tanabe K. (2011). Phase i/ii study of adoptive transfer of gammadelta t cells in combination with zoledronic acid and il-2 to patients with advanced renal cell carcinoma. Cancer Immunol. Immunother..

[38] Santolaria T., Robard M., Leger A., Catros V., Bonneville M., Scotet E. (2013). Repeated systemic administrations of both aminobisphosphonates and human vgamma9vdelta2 t cells efficiently control tumor development in vivo. J. Immunol..

[39] Benzaid I., Monkkonen H., Stresing V., Bonnelye E., Green J., Monkkonen J., Touraine J.L., Clezardin P. (2011). High phosphoantigen levels in bisphosphonate-treated human breast tumors promote vgamma9vdelta2 t-cell chemotaxis and cytotoxicity in vivo. Cancer Res..

[40] Kuo Y.F., Su Y.Z., Tseng Y.H., Wang S.Y., Wang H.M., Chueh P.J. (2010). Flavokawain b, a novel chalcone from alpinia pricei hayata with potent apoptotic activity: Involvement of ros and gadd153 upstream of mitochondria-dependent apoptosis in hct116 cells. Free Radic. Biol. Med..

