# Supplementary material for: Ex Vivo Expanded Human Vγ9Vδ2 T-Cells Can Suppress Epithelial Ovarian Cancer Cell Growth
Source: Int J Mol Sci. 2019 Mar 6;20(5):1139. doi: 10.3390/ijms20051139 (PMC6429417; doi:10.3390/ijms20051139)
Supplement: Supplementary file 1 [file ijms-20-01139-s001.pdf]

Supplemental Materials

3-5-2019 (Version 15)

Table S1: *P*-values comparison between γδ-T only-treated and activated αβ T only-treated cell lines from the figure 2A results.

E/T ratios	0.1	1	10	20
**SKOV3**	n.s.	n.s.	n.s.	n.s.
**HTB75**	n.s.	< 0.0001	< 0.0001	< 0.0001
**OVCAR3**	= 0.0005	< 0.0001	< 0.0001	< 0.0001
**A2780**	n.s.	n.s.	n.s.	n.s.
**A375**	n.s.	n.s.	< 0.0001	< 0.0001
**TOV112D**	n.s.	n.s.	n.s.	n.s.

n.s.: no significance; $^{*}p < 0.05$; $^{**}p < 0.01$; $^{***}p < 0.001$; $^{****}p < 0.0001$

Table S2: *P*-values comparison between γδ-T+PAM-treated and activated αβ T only-treated cell lines from the figure 2A results.

E/T ratios	0.1	1	10	20
**SKOV3**	= 0.0017	< 0.0001	< 0.0001	< 0.0001
**HTB75**	= 0.0356	< 0.0001	< 0.0001	< 0.0001
**OVCAR3**	= 0.0251	= 0.0001	< 0.0001	< 0.0001
**A2780**	= 0.0222	= 0.0119	< 0.0001	< 0.0001
**A375**	= 0.0005	< 0.0001	< 0.0001	< 0.0001
**TOV112D**	= 0.0008	= 0.0001	< 0.0001	< 0.0001

$^{*}p < 0.05$; $^{**}p < 0.01$; $^{***}p < 0.001$; $^{****}p < 0.0001$

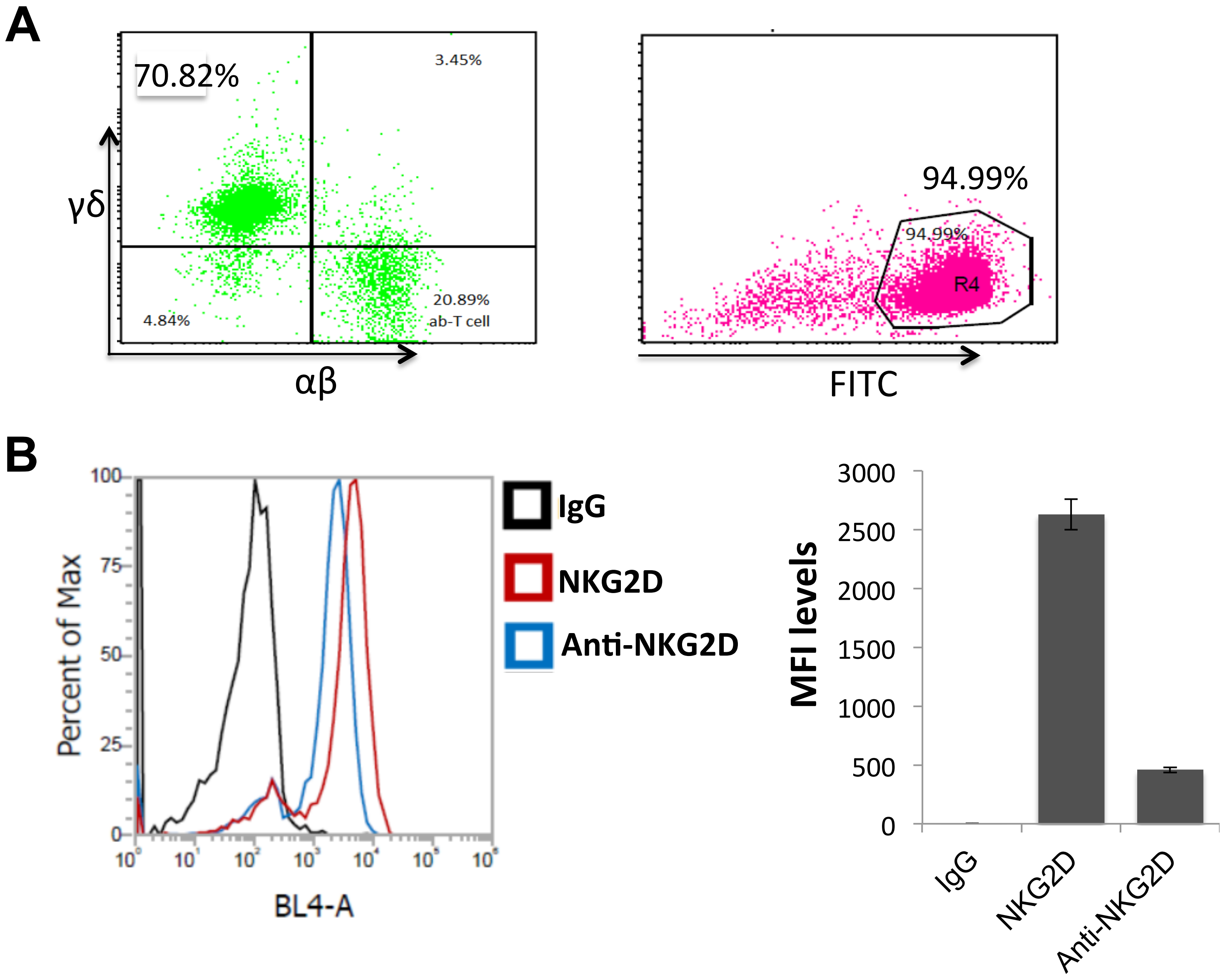

Figure S1: The purity of γδ-T-cell isolation and the efficiency of NKG2D blockade by anti-NKG2D mAb. (A) After a 13-days culture, expanded Vγ9Vδ2 T-cells were at around 70.82% and were purified by positive selection using human anti-TCR γ/δ T-cell MicroBeads according to the manufacture's instructions. There was around 95% purity of γ/δ T-cells following isolation by a flow cytometric analysis. (B) Isolated Vγ9Vδ2 T-cells were incubated with anti-NKG2D mAb before the addition of the indicated tumor target cells. MFI: mean fluorescence intensity.

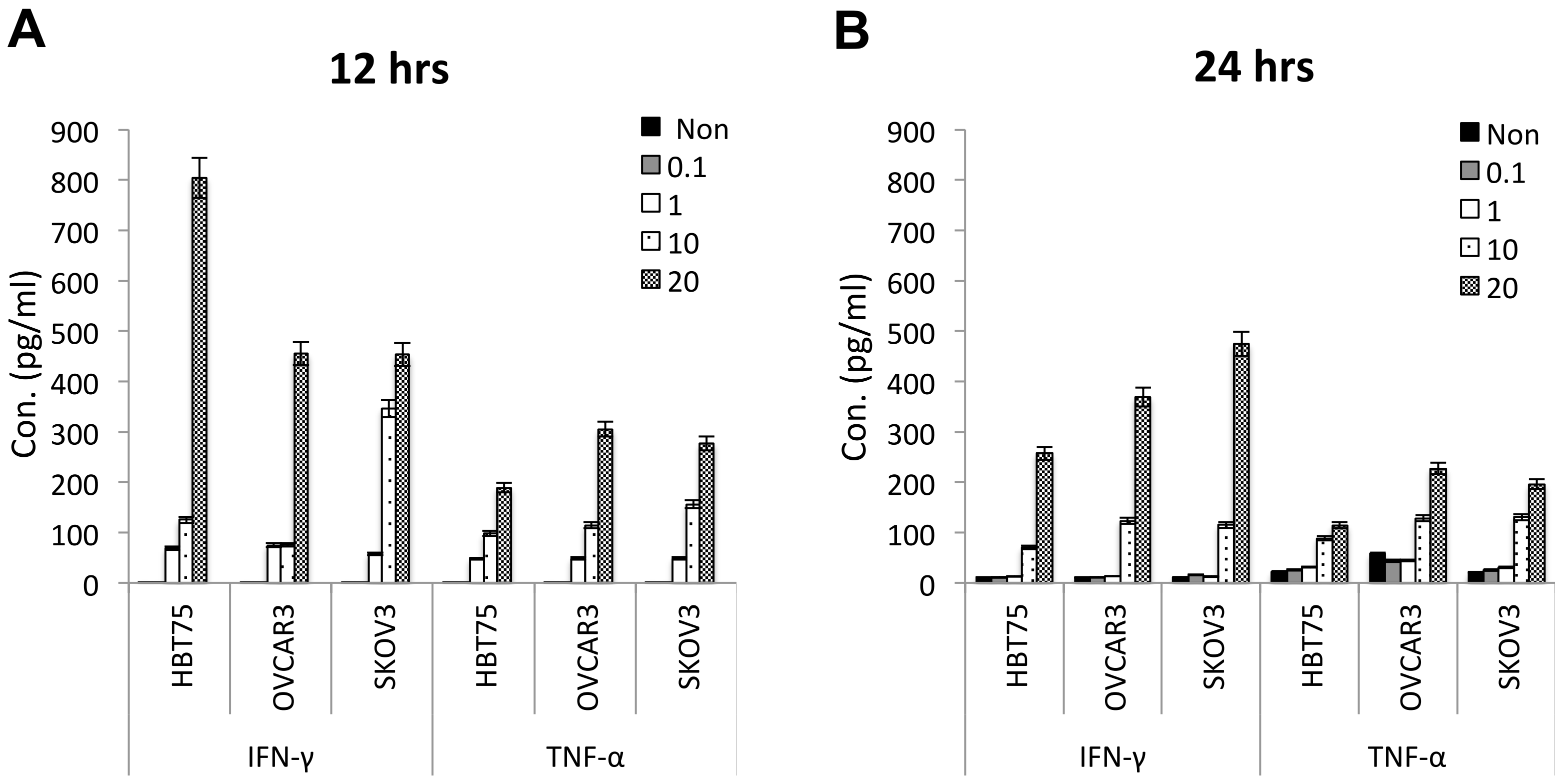

Figure S2. Levels of the cytokines, interferon(IFN)-γ and tumor necrosis factor (TNF)-α, were detected during the γδ-T and tumor cells co-culture cytotoxicity using ELISAs. γδ-T-cell cytotoxicity performed by expansion of γδ-T-cells to tumor cells co-cultured at ratios at 0, 0.1, 1, 10 and 20. Media were harvested for IFN-γ and TNF-α analyses following 12- and 24-h culture periods.

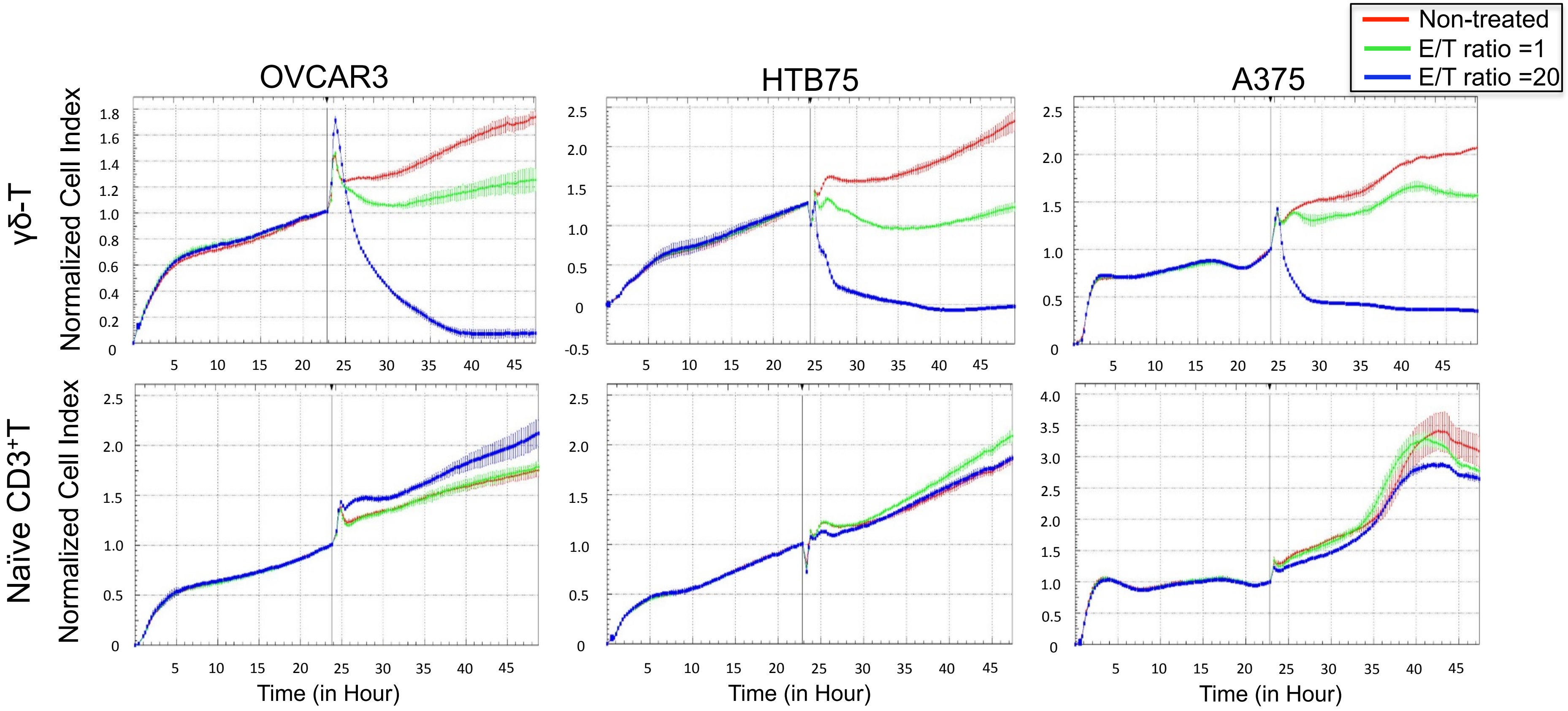

Figure S3: Specific suppression of epithelial ovarian cancer cell lines (HTB75 and OVCAR3), and an epithelial melanoma cell line (A375) by expanded Vγ9Vδ2 T-cells. Standard 24-h cytotoxicity activities were performed with increasing effector (Vγ9Vδ2 T-cells) concentrations to target E/T ratios of 0, 1, and 20 against the cancer cell lines: OVCAR3, HTB75, and A375. Cytotoxic activities were compared to the naïve $CD3^{+}$ T cells served as the controls of Vγ9Vδ2 T-cells. Real-time monitoring of γδ-T cell alone-induced growth inhibition of specific OVCAR3, HTB75, and A375 cells using the x-CELLigence system. Data are presented as the mean ± SD of three independent experiments.

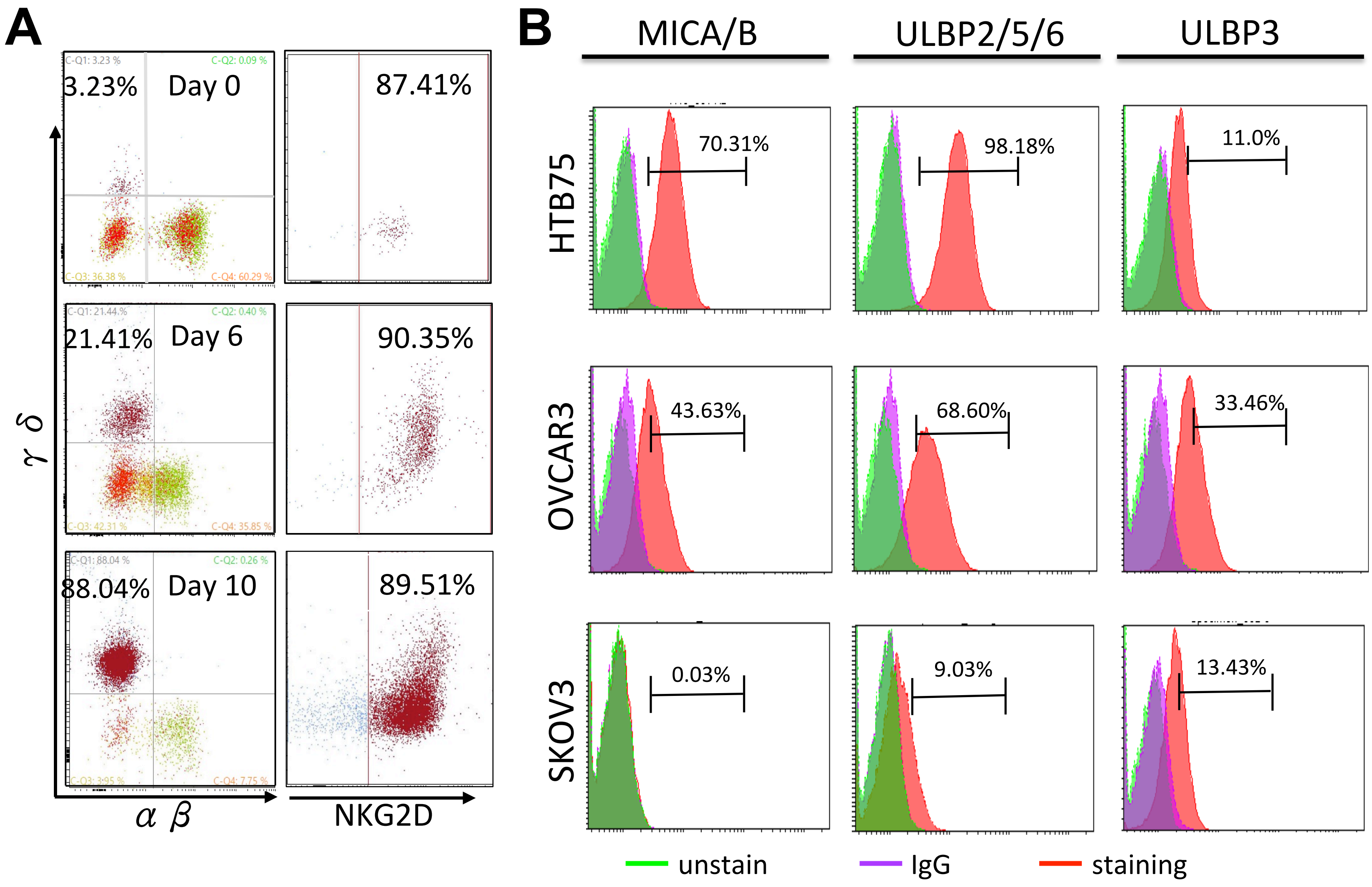

Figure S4: Receptor-ligand interactions mediating ovarian cancer cell recognition by expanded Vγ9Vδ2-T-cell-targeted cytotoxicity. (A) On days 0, 6, and 10 of the expansion period, the γδ-T expanded were at 3.23%, 21.41% and 88.04%, respectively (A, left panel), whereas around 90% of the expanded Vγ9Vδ2-T-cells showed NKG2D expression (A, right panel). (B) NKG2D ligand (NKG2DL) expression was measured in the ovarian cancer cell lines: HTB75, OVCAR3, and SKOV3. MICA/B, ULBP2/5/6, and ULBP-3 were strongly expressed by epithelial-type HTB75 and OVCAR3 cells compared to non-epithelial-type SKOV3 cells. Data are presented as the mean ± SD of at least three independent experiments.
